# Serum Lactate Dehydrogenase to Phosphate Ratio as an Independent Predictor for Adverse Outcome of Microsurgical Clipping for Ruptured Intracranial Aneurysm: A Propensity-Score Matching Analysis

**DOI:** 10.3390/brainsci12060737

**Published:** 2022-06-04

**Authors:** Shufa Zheng, Yibin Zhang, Haojie Wang, Xueling Xie, Yuanxiang Lin, Peisen Yao, Zhangya Lin, Dezhi Kang

**Affiliations:** 1 Department of Neurosurgery, Neurosurgery Research Institute, The First Affiliated Hospital, Fujian Medical University, Fuzhou, 350004, China; zsf2002110@163.com (S.Z.); eabin.z@fjmu.edu.cn (Y.Z.); haojie2016@fjmu.edu.cn (H.W.); fjmu1234@sina.com (X.X.); lyx99070@163.com (Y.L.); peisen_yao@fjmu.edu.cn (P.Y.); 13799321745@139.com (Z.L.); 2 Fujian Provincial Clinical Research Center for Neurological Disease, the First Affiliated Hospital, Fujian Medical University, Fuzhou, 350004, China; 3 Department of Neurosurgery, Binhai Branch of National Regional Medical Center, the First Affiliated Hospital, Fujian Medical University, Fuzhou, 350004, China; 4 Fujian Provincial Institutes of Brain Disorders and Brain Sciences, the First Affiliated Hospital, Fujian Medical University, Fuzhou, 350004, China; 5 Clinical Research and Translation Center, The First Affiliated Hospital, Fujian Medical University, Fuzhou, 350004, China; 6 The First Affiliated Hospital, Fujian Medical University, No. 20 Chazhong Road, Taijiang District, Fuzhou 350004, China

**Keywords:** aneurysmal subarachnoid hemorrhage, lactate dehydrogenase, phosphate, biomarker, outcome, microsurgical clipping

## Abstract

Objective: In this study, we assessed the correlation between the lactate dehydrogenase (LDH) to phosphate ratio and the prognosis of microsurgical clippings for ruptured intracranial aneurysm (rIA) to test the hypothesis that the serum LDH to phosphate ratio could be a predictor of the outcome of microsurgical clipping for rIA. Methods: Records of rIA patients between 2012 and 2018 were retrospectively collected. Age, sex, Hunt-Hess grade, Fisher grade, medical history, aneurysm location, hydrocephalus, laboratory data including serum LDH, phosphate, and LDH to phosphate ratio, related complications, and the outcomes in 3 months were recorded. Results: A total of 1608 rIA patients in our institution were collected, and 856 patients treated by microsurgical clipping were enrolled. On admission, a significantly higher LDH-phosphate ratio was observed in patients with poor outcomes at 3 months (median ± SD, 200.175 ± 107.290 for mRS 0–2 vs. 323.826 ± 219.075 for mRS score 3–6; *p* = 0.000). An LDH to phosphate ratio of 226.25 in the receiver operating characteristic (ROC) curve was the optimal cutoff value to discriminate between good and poor outcomes at 3 months. The LDH to phosphate ratio ≥ 226.25 on admission was independently correlated with poor outcomes in rIA patients. In addition, Hunt and Hess grade, Fisher grade, pneumonia, and DIND were also independently correlated with poor outcomes. After removing the bias in essential clinical variables between patients with LDH to phosphate, ratio ≥ 226.25 versus <226.25 by PSM, the number of patients with poor outcomes at 3 months increased in patients with an LDH to phosphate ratio of ≥226.25 (*p* = 0.005). Conclusions: The LDH to phosphate ratio was a potential biomarker and could predict the unfavorable outcome of microsurgical clipping for rIA in 3 months, related to neuronal damage, cerebral hypoxia, and early brain injury after aneurysm ruptures.

## 1. Introduction

It was revealed that neuronal damage and brain tissue hypoxia were the two main mechanisms involved in early brain injury after subarachnoid hemorrhage [1,2]. Many serum nonspecific biomarkers, such as C-reactive protein (CRP) [3,4], serum interleukin-6 [5,6], neutrophil-lymphocyte ratio [7], serum glucose level [8,9], serum glucose/potassium ratio [10], and serum glucose/phosphate ratio [11], were risk factors for predicting the unfavorable prognosis of ruptured intracranial aneurysm (rIA). Some of them might be involved in the mechanism of early brain injury following an aneurysmal subarachnoid. However, the process and detailed mechanism of early brain injury in rIA is multifactorial and remains unclear.

Lactate dehydrogenase (LDH) is a glycolytic enzyme in brain tissue and can be released from the damaged neuronal cells when the cell membrane is destroyed, which is nonspecific and exists in other human organs, including the liver, heart, skeletal muscle, kidney, and lung. It was reported that LDH could predict the unfavorable outcome of traumatic brain injury [12,13,14], intracerebral hemorrhage [15], and aneurysmal subarachnoid hemorrhage [16], but few studies assessed the relationship between LDH and the outcome of rIA. Brain tissue hypoxia could be induced by hypophosphatemia because it can promote an adverse increase in hemoglobin oxygen affinity and reduce the oxygen release to the brain [17], resulting in secondary cerebral hypoxia and brain tissue injury. It was established that hypophosphatemia is a biomarker for the severity of illness in an intensive care unit [18,19]. However, hypophosphatemia was correlated with the prognosis of aneurysmal subarachnoid hemorrhage [11,20]. Hypophosphatemia and elevated LDH may be closely related in patients with rIA. Here, we tested the hypothesis that serum LDH and phosphate might be associated with neuronal damage, cerebral hypoxia, and early brain injury and assessed the correlation between the LDH to phosphate ratio and the prognosis of rIA, which may be a predictor of the adverse outcome of rIA.

## 2. Methods

The data were retrospectively retrieved from a prospectively collected database. All procedures performed in this retrospective study involving human participants were based on the 1964 Helsinki declaration and approved by the ethics committee of First Affiliated Hospital of Fujian Medical University.

## 3. Patient Characteristics

Patients were enrolled in the study based on the following criteria: (1) Aneurysmal subarachnoid hemorrhage (aSAH) was diagnosed by computed tomography (CT) and computerized tomography angiography (CTA) or digital subtraction angiography (DSA). (2) The patients were admitted 24 h after the occurrence of SAH. (3) Cerebral aneurysms were treated by microsurgical clipping. The exclusion criteria were: (1) aSAH detected > 24 h after the onset. (2) The presence of intracranial tumors in patients with other cerebrovascular diseases (such as intracranial arteriovenous malformations, arteriovenous fistula, and Moyamoya syndrome/disease). (3) Patients with myocardial infarction, pulmonary infarction, hepatitis, kidney disease or progressive muscular atrophy, malignant tumor, leukemia, hemolytic anemia, etc.

Essential clinical characteristics of rIA patients include age, sex, H-H grade, Fisher grade, smoking, drink, medical history, aneurysm location, hydrocephalus, laboratory data (white blood cells, hemoglobin, serum glucose, serum LDH, serum phosphate, and LDH to phosphate ratio), complication (intracranial hematoma, intracranial infection, pneumonia, sepsis and delay ischemic neurological deficit), and the outcomes in 3 months are shown in Table 1. Upon admission, all the patients had peripheral venous blood drawn for laboratory examinations, including their blood routine and biochemical blood indexes.

**Figure 1:**
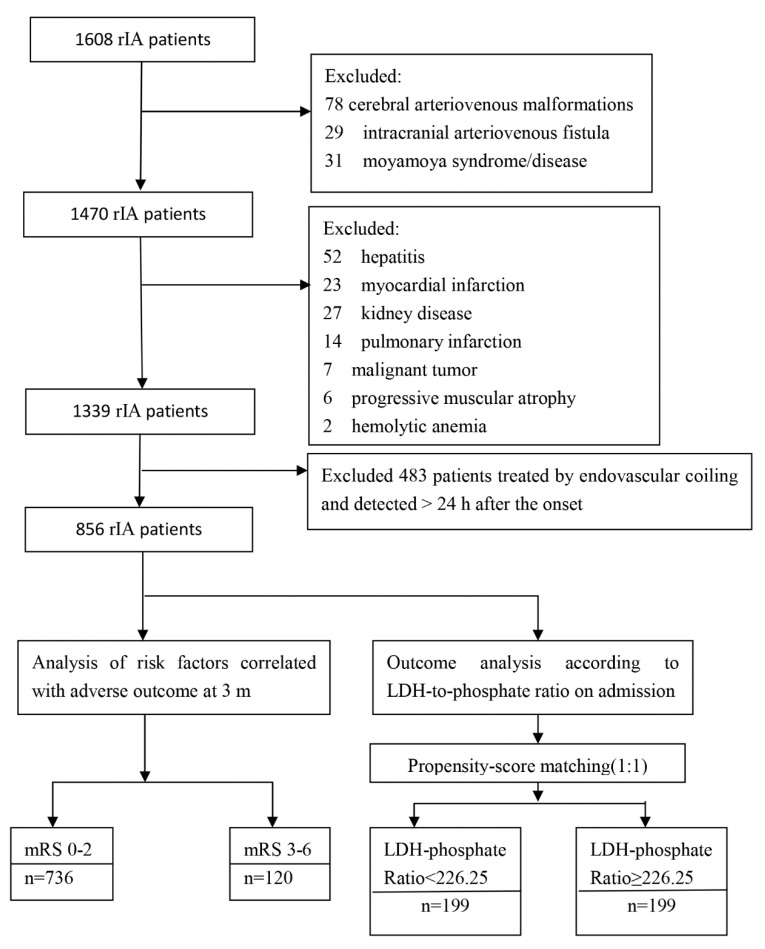
Flowchart of the study.

The records of a total of 1608 rIA patients in our institution were collected, and 856 patients treated by microsurgical clipping were enrolled in our institution between 2012 and 2018 (shown in Figure 1 and Table 1). The patients included 337 (39.4%) males and 519 (60.6%) females, and the mean age was 54.5 years (range 10–86). The mean ± SD of white blood cell, (×10^9^/L) was 10.009 ± 4.347; serum glucose (mmol/L), 6.580 ± 3.155; serum LDH (U/L), 186.914 ± 58.595; serum phosphate (mmol/L), 0.992 ± 0.348; serum LDH- phosphate ratio, 217.509 ± 135.722. The time from intracranial aneurysm rupture to admission ranged from 1 h to 19 days (mean time 30.6 ± 18.1 h).

## 4. Preoperative Management

All rIA patients receive CT scans routinely on admission to our institution. Clinical condition and the image characteristics were evaluated in all rIA patients using H-H grade and Fisher grade. CTA or DSA examinations were performed to diagnose the cerebral aneurysm. The cerebral aneurysm was treated by microsurgical clipping 3 days after admission. Perioperative care and medical treatment were implemented according to a standardized protocol and the guidelines for managing aneurysmal subarachnoid in China. Postoperative complications were evaluated by CT scanning within 24 h after microsurgical clipping. All patients underwent CTA or DSA examination within postoperative day 7 to confirm whether there existed a cerebral vasospasm and residual aneurysm neck or not.

The 3-month outcome was evaluated using the Modified Rankin Scale (mRS). An mRS score of 0–2 was defined as a good outcome, and an mRS score of 3–6 was defined as a poor outcome. CT or MR imaging confirmed a delayed ischemic neurological deficit as the appearance of clinical symptoms, such as newly developed focal neurological deficits or loss of consciousness.

## 5. Statistical Analysis

Statistical analyses were carried out with SPSS for windows (version 25.0, IBM Corp., Armonk, NY, USA), GraphPad Prism (version 8.3.0, GraphPad Software, San Diego, CA, USA) and Medcal software (version 20.0.4, Mariakerke, Belgium). The differences in the continuous variables were evaluated using the Student’s *t*-test or one-way analysis of variance (ANOVA). The differences in qualitative variables were determined using the Chi-squared test (χ^2^ test) or Fisher’s exact test. The multivariable analysis included all variables with a *p*-value of less than 0.10 in a univariate analysis. The correlations between serum LDH to phosphate ratio and H-H grade, serum LDH and H-H grade, serum phosphate and H-H grade, serum LDH, and serum phosphate were evaluated utilizing Spearman’s rank correlation coefficient. The serum LDH to phosphate ratio between the good outcome group (mRS score 0–2) and poor outcome group (mRS score 3–6) were evaluated using Mann–Whitney U-tests. Differences were deemed significant at a *p* < 0.05. The receiver operating curve (ROC) evaluated the specificity and sensitivity of serum LDH to a phosphate ratio for a 3-month outcome. A propensity-score matching (PSM) analysis was carried out to remove imbalances in essential clinical variables between the serum LDH to phosphate Ratio ≥optimal cutoff value and <optimal cutoff value group. Conditional probability was estimated with the logistic regression model. The serum LDH to phosphate ratio ≥optimal cutoff value and <optimal cutoff value groups were matched at a ratio of 1:1 using the nearest neighboring matching algorithm.

## 6. Results

### 6.1. The Primary Clinical Characteristics of rIA Patients

The essential characteristics of patients with rIA are shown in Table 1. Seven hundred and thirty-six patients had a favorable outcome compared to 120 with unfavorable outcomes in the present study. The clinical characteristics of the two groups are shown in Table 2. There existed a significant difference in age, H-H grade, Fisher grade, hydrocephalus, white blood cells, serum glucose, serum LDH, serum LDH to phosphate ratio, pneumonia, and the delayed ischemic neurological deficit between the two groups (shown in Table 2). The rIA Patients with poor outcomes at 3 months were older (*p* = 0.003), had a poor Hunt-Hess grade (*p* < 0.001), poor grade Fisher grade (*p* < 0.001), and a higher rate of pneumonia (*p* < 0.001) and delayed ischemic neurological deficit (*p* < 0.001). The present study’s mean ± SD of white blood cells (×10^9^/L) was 10.009 ± 4.347. Higher WBC on admission was observed in patients with poor outcomes at 3 months (*p* = 0.000; Table 2). A significantly higher LDH on admission was observed in patients with poor outcomes at 3 months (*p* = 0.000, shown in Table 2). The prevalence of hypophosphatemia (<0.80 mmol/L) was 26.3% (225/856). Moreover, there is a statistical difference in serum phosphate levels between the two groups (*p* < 0.001, shown in Table 2). Interestingly, a significantly higher serum LDH-phosphate ratio on admission was observed in patients with poor outcomes at 3 months (median ± SD, 200.175 ± 107.290 for mRS 0–2 vs. 323.826 ± 219.075 for mRS score 3–6; *p* = 0.000; see Table 2).

### 6.2. The Correlation of LDH- Phosphate Ratio with the Functional Outcome of rIA Patients

**Figure 2:**
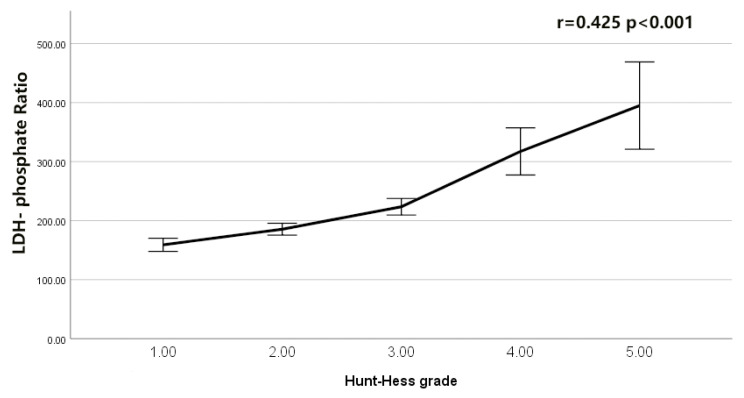
Correlation of LDH−phosphate Ratio with Hunt−Hess grade after intracranial aneurysm rupture.

**Figure 3:**
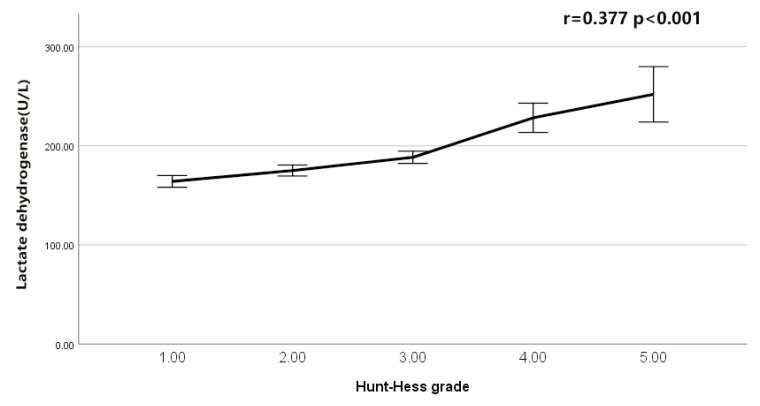
Correlation of LDH with Hunt−Hess grade after intracranial aneurysm rupture.

**Figure 4:**
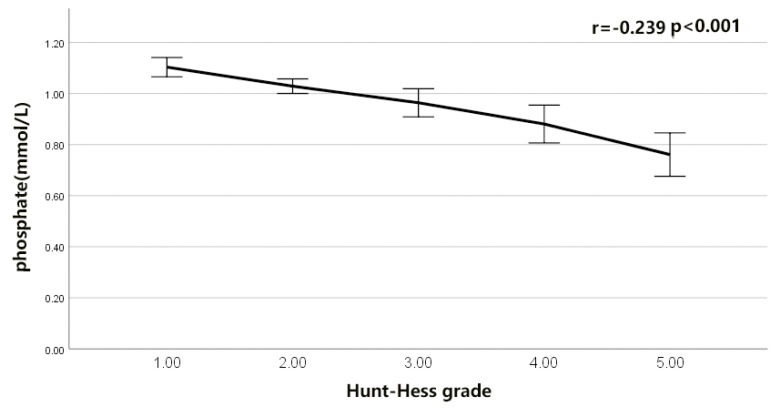
Correlation of serum phosphate with Hunt−Hess grade after intracranial aneurysm rupture.

**Figure 5:**
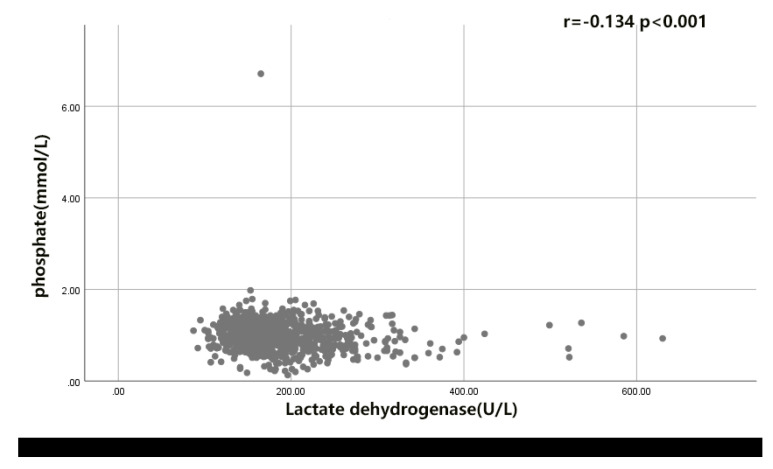
Correlation of LDH with phosphate after intracranial aneurysm rupture.

**Figure 6:**
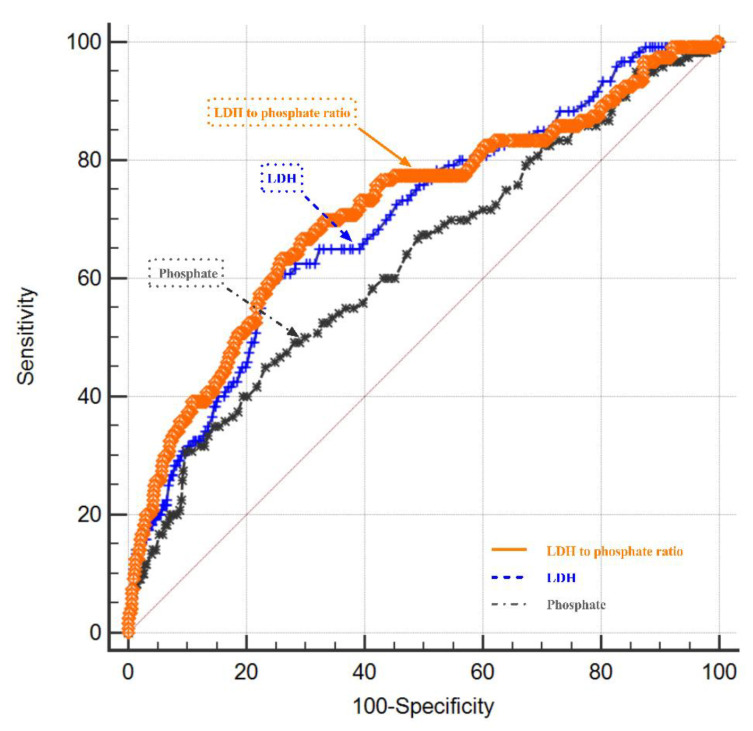
ROC curve of the three predictors. Predictive values of LDH-phosphate Ratio for 3-month modified Rankin Scale (mRS) >2 area under curve 0.713 (the cutoff = 226.25, 95% CI, 0.681–0.743; *p* < 0.001; the sensitivity was 63.33%, and the specificity was 74.18%). The AUC of LDH and phosphate were 0.698 (95% CI 0.666–0.729, *p* < 0.001; the sensitivity was 59.17%, and the specificity was 75.95%), 0.630 (95% CI 0.597–0.662, *p* < 0.001; the sensitivity was 45.00%, and the specificity was 76.77%), respectively.

The serum LDH to phosphate ratio was elevated with an increasing H-H grade (Spearman’s r = 0.425, *p* < 0.001; Figure 2). Serum LDH was also elevated with an increasing H-H grade (r = 0.377, *p* < 0.001; Figure 3). The serum phosphate level decreased with an increasing H-H grade (r = −0.239, *p* < 0.001; Figure 4). The serum phosphate level decreased with an increase in the serum LDH level (r = −0.134, *p* < 0.001; Figure 5). Multivariable Logistic regression analyses demonstrated that the H-H grade, Fisher grade, serum LDH to phosphate Ratio ≥ 226.25, pneumonia, and DIND were independently associated with the unfavorable outcome of rIA (*p* < 0.05). The results showed that the serum LDH to phosphate ratio on admission was independently correlated with poor outcomes in rIA patients (risk ratio [95% CI] 1.967 [1.185–3.266] *p* = 0.009). In addition, Hunt and Hess grade (risk ratio [95% CI] 1.731 [1.333–2.246] *p* < 0.001), Fisher grade (risk ratio [95% CI] 1.428 [1.108–1.842] *p* = 0.0096), pneumonia (risk ratio [95% CI] 4.017 [2.472–6.530] *p* < 0.001) and DIND (risk ratio [95% CI] 3.773 [2.171–6.559] *p* < 0.001) were also independently correlated with poor outcome (Table 3). The receiver operating characteristic (ROC) curve of serum LDH to the phosphate ratio for poor outcomes of rIA patients is shown in Figure 6. A serum LDH-phosphate ratio of 226.25 was identified as the optimal cutoff value to discriminate between good and poor outcomes at 3 months. The area under the ROC curve (AUC) was 0.713 (95% CI, 0.681–0.743; the sensitivity was 63.33%, and the specificity was 74.18%, *p*< 0.001) (mRS 3–6: LDH to phosphate ratio ≥226.25 267/856 [31.2%] vs. LDH- phosphate ratio < 226.25 589/856 [68.8%]; *p* < 0.001). The ROC analysis also revealed that the AUC was 0.698 (95% CI 0.666–0.729, *p* < 0.001; the sensitivity was 59.17%, and the specificity was 75.95%) for LDH and 0.630 (95% CI 0.597–0.662, *p* < 0.001; the sensitivity was 45.00%, and the specificity was 76.77%) for phosphate, respectively (Figure 6). The Z-test illustrated that the AUC of LDH to phosphate ratio was statistically higher than the phosphate (Z = 4.892, *p* < 0.001).

### 6.3. The Outcomes of rIA Patients in PSM Analysis

**Figure 7:**
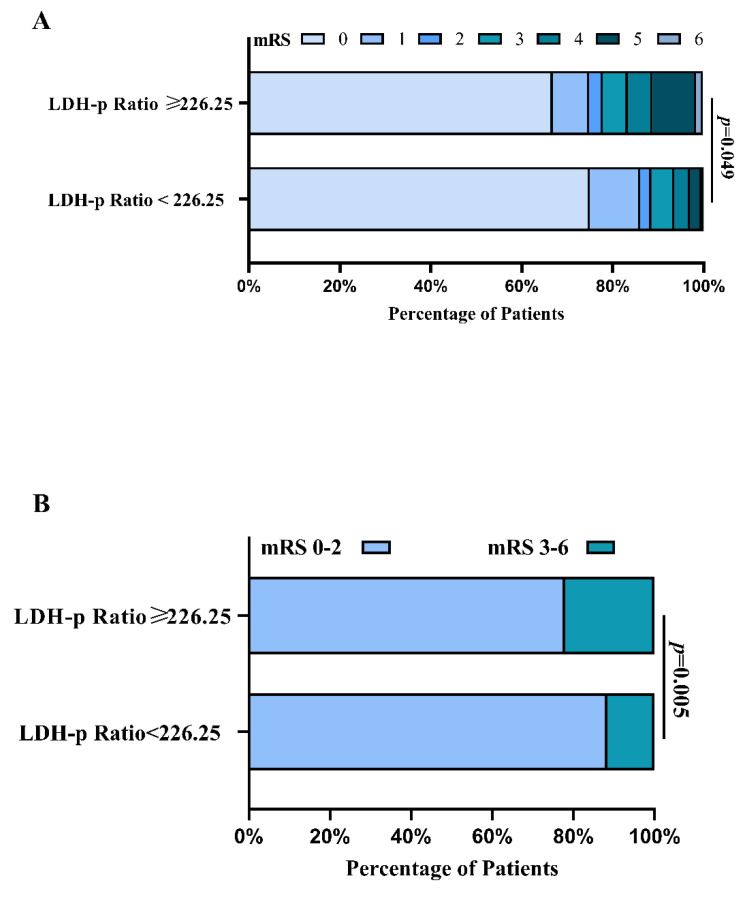
The mRS scores at 3 months for rIA patients with LDH to phosphate ratio ≥ 226.25 versus < 226.25 after PSM. (see Table 3 for a detailed description). (**A**). Proportions of patients within each mRS score category on the 7-point scale (where o indicates no symptoms and 6 indicates death) at 3 months between patients with LDH to phosphate ratio ≥ 226.25 versus < 226.25. (**B**). Distribution of functional outcomes at 3 months between patients with LDH to phosphate ratio ≥ 226.25 versus < 226.25.

rIA Patients with a serum LDH to phosphate ratio ≥ 226.25 were more prone to an unfavorable clinical condition on admission (H-H grade) (Figure 1). To remove bias in basic clinical variables between patients with an LDH to phosphate ratio ≥ 226.25 versus <226.25, PSM was performed; finally, it was revealed (Table 4) that there were no differences in age (*p* = 0.806), HH grade (*p* = 0.130), Fisher (*p* = 1.000), pneumonia (*p* = 0.913) and DIND (*p* = 0.538). Figure 7 illustrates the functional outcome of 3-month mRS in patients with a serum LDH to phosphate ratio ≥ 226.25 versus <226.25. The comparison of the proportions of patients within each mRS score category on the 7-point scale at 3 months between the two groups is presented in Figure 7A. Compared to the patients with a serum LDH to phosphate ratio < 226.25, the proportions of patients with unfavorable outcomes at 3 months increased in patients with a serum LDH to phosphate ratio ≥ 226.25 (*p* = 0.005; shown in Figure 7B).

## 7. Discussion

This study intended to assess the role of the serum LDH to phosphate ratio in the clinical conditions and 3-month outcomes in rIA patients. Here, we firstly explored the predictive value of LDH to the phosphate ratio in rIA patients. It was demonstrated that rIA patients with a serum LDH to phosphate ratio ≥ 226.25 were more prone to an unfavorable clinical condition on admission (H-H grade), and a significantly higher serum LDH-phosphate ratio on admission was observed in patients with poor outcomes at 3 months. Moreover, a LDH-phosphate ratio ≥ 226.25 on admission was independently correlated with poor outcomes in rIA patients. In addition, the Hunt and Hess grade, Fisher grade, pneumonia, and DIND were also independently correlated with poor outcomes, and these results were consistent with the previous study [21]. After removing the bias in essential clinical variables between patients with a serum LDH to phosphate ratio ≥ 226.25 versus <226.25 by PSM, the number of patients with poor outcomes at 3 months increased in patients with a serum LDH to phosphate ratio ≥ 226.25 (*p* = 0.005), suggesting that the serum LDH to phosphate ratio is a potential biomarker that can reflect clinical conditions and predict unfavorable outcomes in rIA patients in 3 months.

As Lu Y reported, the number of damaged cells was positively related to the clinical condition of rIA patients and their Hunt and Hess grade [22]. After the intracranial aneurysm ruptures, cytolysis occurs, or the neuronal cell membrane is destroyed, and LDH will be released into the blood, resulting in an increase in serum LDH [23]. Hence, the following factors resulted in elevated serum LDH levels in rIA patients: (1) LDH originating from the damaged neuron or glial cells. (2) LDH produced from lytic red blood cells (RBC) after being released into the subarachnoid space. As we know, LDH can catalyze the dehydrogenation of lactic acid to pyruvic acid, promote anaerobic glycolysis, and prevent lactic acid accumulation, the latter of which is correlated with poor outcomes of traumatic brain injury [14]. Therefore, we inferred that cerebral hypoperfusion was prone to occur in rIA patients, especially those with severe conditions, which would lead to cerebral hypoxia and lactic acid accumulation. The latter might promote the elevation of serum LDH levels. Several reports demonstrated that the serum LDH level was correlated with the prognosis of neuroblastoma [24], glioblastoma multiforme [25], acute encephalopathy [26], and mycoplasma pneumonia [27]. Yu W’s study revealed that the serum LDH level was associated with the degree of brain tissue injury, and serum LDH activities were correlated with cerebral artery occlusion in a dose-dependent manner [28]. Several reports also showed that the LDH level was used to predict neuronal injury [29,30], and could also be a predictor of the unfavorable outcome of traumatic brain injury [31] and neonatal intracranial hemorrhage [15,32]. Interestingly, our present study showed that serum LDH was also elevated with an increasing H-H grade (*p* < 0.001), which may be a nonspecific biomarker of damaged brain tissue after an aneurysm ruptures.

Acute spontaneous intracerebral hemorrhage, including aSAH, is accompanied by hypophosphatemia [33,34]. Accordingly, these reasons explain the underlying mechanism. Firstly, patients with aSAH are prone to spontaneous hyperventilation [33]. As a result of respiratory alkalosis, the body’s pH changes, which then causes a drop in phosphate levels due to a series of metabolic reactions [11,35]. Hypophosphatemia occurs when phosphate is transferred into the cells. Patients with aSAH are likely to develop hypophosphatemia due to this condition. Secondly, elevated serum endogenous or exogenous catecholamines (e.g., epinephrine and norepinephrine) can negatively affect serum phosphate in patients with aSAH [11,36]. Finally, inflammatory factors lead to the internal redistribution of phosphate, which might explain the decrease in serum phosphate [5,6].

After aSAH, cerebral hypoxia participates in the early brain injury [1,2]. However, the detailed mechanism of early brain injury remains unclear. It is known that phosphate is essential to produce 2,3-diphosphoglycerate in red blood cells, and low phosphate levels hamper the production of 2,3-diphosphoglycerate, which can incur an adverse increase in hemoglobin oxygen affinity and reduces the oxygen release to the brain [17]. Ultimately, brain energy metabolism is impaired, and brain tissue injury occurs. Suzuki’s studies demonstrated that hypophosphatemia was associated with illness severity and higher ICU mortality [20]. Our present study showed that the prevalence of hypophosphatemia was 26.3% in rIA patients, which was in rough agreement with the previous study [20]. Our results also showed that the serum phosphate level of the rIA patients decreased with the increase in the H-H grade (r = −0.239, *p* < 0.001).

Furthermore, the serum phosphate level in the unfavorable group was significantly lower than in the favorable group. Therefore, hypophosphatemia may participate in early brain injury by reducing oxygen release to brain tissue. Here, we assumed that lower serum phosphorus was a risk factor and was prone to causing cerebral hypoxia. Hypophosphatemia was associated with the adverse prognosis of rIA.

Interestingly, when we introduced the serum LDH to phosphate ratio into one of the risk factors, we found that spearman’s r value of LDH to phosphate ratio is higher than that of serum LDH and serum phosphate level. It suggested that serum LDH to phosphate ratio is more suitable as a predictor for clinical conditions and poor outcomes of rIA patients than serum LDH and serum phosphate. Here, we also found that serum phosphate levels decreased with increasing serum LDH levels (r = −0.134, *p* < 0.001) in the present study, but the detailed mechanism remains unclear and requires further exploration.

## 8. Limitations

As this was a retrospective study, there were several limitations. First, the serum LDH to phosphate ratio lacks specificity to brain tissue. The LDH from CSF was not collected. The serum LDH level cannot directly reflect the accurate level in the brain tissue. Future prospective multicenter studies based on the LDH to phosphate ratio in CSF samples are warranted. Second, the time from rupture to the time of collection of serum LDH and phosphate was not consistent. Third, our results were only based on the patients treated by microsurgical clipping, and it was necessary to clarify whether this conclusion is suitable for patients treated by endovascular coiling. Fourth, it is a retrospective study and not a prospective and multicenter design. Although PSM was performed to remove the imbalance of clinical variables, our study showed that associations of serum LDH to the phosphate ratio were correlated with unfavorable outcomes in rIA patients; and the detailed mechanism remains unknown. Large-scale randomized clinical trials are needed to further confirm the conclusion.

## 9. Conclusions

Serum LDH to phosphate ratio is a potential biomarker and could predict the poor outcome of microsurgical clipping for rIA in 3 months. LDH and phosphate may be related to neuronal damage, cerebral hypoxia, and early brain injury after aneurysm ruptures. However, the detailed mechanism remains unclear, and large-scale randomized clinical trials should be conducted to further confirm the conclusion.

## Figures and Tables

**Table 1:** Patient characteristics.

Variable	Value
No. of patients	856
Mean age in years (range)	54.5 (10–86)
Female	519 (60.6)
Smoking	178 (20.8)
Drink	85 (9.9)
Medical history	
Hypertension	379 (44.3)
Diabetes	50 (5.8)
Coronary heart disease	11 (1.3)
Cerebral stroke	15 (1.8)
aneurysm location	
anterior circulation	694 (81.1)
posterior circulation	9 (1.1)
multiple aneurysm	153 (17.9)
Hunt-Hess grade	
I	145 (16.9)
II	313 (36.6)
III	264 (30.8)
IV	88 (10.3)
V	46 (5.4)
Fisher	
1	250 (29.2)
2	244 (28.5)
3	171 (20.0)
4	191 (22.3)
Hydrocephalus	168 (19.6)
Laboratory values	
White blood cell, ×109/L, mean ± SD	10.009 ± 4.347
Hemoglobin (g/L)	128.627 ± 18.680
Glucose (mmol/L), mean ± SD	6.580 ± 3.155
Lactate dehydrogenase (U/L), mean ± SD	186.914 ± 58.595
serum phosphate (mmol/L), mean ± SD	0.992 ± 0.348
LDH-phosphate Ratio, mean ± SD	217.509 ± 135.722
Complication	
Postoperative intracranial hematoma	71 (8.3)
Postoperative intracranial infection	66 (7.7)
Postoperative Pneumonia	220 (25.7)
Sepsis	12 (1.4)
Delay ischemic neurological deficit	115 (13.4)

**Table 2:** Univariate analysis of the risk factor of ruptured intracranial aneurysm for poor outcomes at 3 months.

General Information (*n* = 856)	Good Outcome	Poor Outcome	*p*−Value
	(*n* = 736)	(*n* = 120)	
Mean age in years (range)	54.1 (10–86)	57.4 (22–85)	0.003
Female	439 (59.6)	80 (66.7)	0.144
Smoking	148 (20.1)	30 (25.0)	0.221
Drink	75 (10.2)	10 (8.3)	0.528
Medical history			
Hypertension	317 (43.1)	62 (51.7)	0.079
Diabetes	39 (5.3)	11 (9.2)	0.094
Coronary heart disease	9 (1.2)	2 (1.7)	0.689
Cerebral stroke	13 (1.8)	2 (1.7)	0.939
Aneurysm location			0.177
Anterior circulation	604 (82.1)	90 (75.0)	
Posterior circulation	7 (1.0)	2 (1.7)	
Multiple aneurysms	125 (17.0)	28 (23.3)	
Hunt−Hess grade			<0.001
I	138 (18.8)	7 (5.8)	
II	293 (39.8)	20 (16.7)	
III	233 (31.7)	31 (25.8)	
IV	54 (7.3)	34 (28.3)	
V	18 (2.4)	28 (23.3)	
Fisher grade			<0.001
1	240 (32.6)	10 (8.3)	
2	229 (31.1)	15 (12.5)	
3	139 (18.9)	32 (26.7)	
4	128 (17.4)	63 (52.5)	
Hydrocephalus	115 (15.6)	53 (44.2)	<0.001
Lab values			
White blood cell, ×109/L, mean ± SD	9.634 ± 4.033	12.307 ± 5.394	<0.001
Hemoglobin (g/L)	128.523 ± 18.252	129.267 ± 21.190	0.686
Glucose (mmol/L), mean ± SD	6.360 ± 3.122	7.928 ± 3.024	0.008
Lactate dehydrogenase (U/L), mean ± SD	180.378 ± 50.695	227 ± 83.125	<0.001
Serum phosphate (mmol/L), mean ± SD	1.012 ± 0.347	0.863 ± 0.319	<0.001
LDH−phosphate Ratio, mean ± SD	200.175 ± 107.290	323.826 ± 219.075	<0.001
Complication			
Postoperative intracranial hematoma	61 (8.3)	10 (8.3)	0.987
Postoperative intracranial infection	53 (7.2)	13 (10.8)	0.167
Postoperative Pneumonia	142 (19.3)	78 (65.0)	<0.001
Sepsis	10 (1.4)	2 (1.7)	0.790
Delay ischemic neurological deficit	72 (9.8)	43 (35.8)	<0.001

The rIA patients were categorized according to the 3-month outcome (mRS score 0–2 vs. 3–6). Values are the number of patients (%) or median ± SD or median (range).

**Table 3:** Multivariate analysis of risk factors associated with a poor outcome at 3 months.

Variable	Adjusted OR	95% CI	*p*-Value
Age	1.016	0.996–1.037	0.118
Hunt-Hess grade	1.731	1.333–2.246	<0.001
Fisher	1.428	1.108–1.842	0.006
Hydrocephalus	1.090	0.648–1.833	0.747
White blood cell	0.979	0.926–1.035	0.446
Glucose	1.025	0.964–1.089	0.432
LDH- phosphate Ratio ≥ 226.25	1.967	1.185–3.266	0.009
Pneumonia	4.017	2.472–6.530	<0.001
Delay ischemic neurological deficit	3.773	2.171–6.559	<0.001

**Table 4:** Characteristics of rIA patients dichotomized to the LDH-phosphate ratio threshold (226.25) before and after PS matching.

General Information	LDH−Phosphate Ratio on Admission	LDH−Phosphate Ratio on Admission	*p*-Value
	≥226.25	<226.25	
Pre–PS match			
No. of patients	267(30.9)	589 (68.1)	
Mean age in years (range)	55.9 (25–85)	53.9 (10–86)	0.016
Hunt–Hess grade			
I–III	174(65.2)	548(93.0)	<0.001
IV–V	93(34.8)	41 (7.0)	
Fisher			
1–3	161 (60.3)	506 (85.9)	<0.001
4	106 (39.7)	85 (14.4)	
Hydrocephalus	85 (31.8)	83 (14.1)	<0.001
Lab values			
White blood cell, ×10^9^/L, mean ± SD	12.208 ± 4.728	9.011 ± 3.764	<0.001
Glucose (mmol/L), mean ± SD	7.506 ± 2.574	6.159 ± 3.302	<0.001
Lactate dehydrogenase (U/L), mean ± SD	233.004 ± 72.611	166.020 ± 34.718	<0.001
LDH–phosphate Ratio, mean ± SD	357.445 ± 166.110	154.075 ± 37.147	<0.001
Complication			
Pneumonia	113 (42.3)	107 (18.2)	<0.001
Delay ischemic neurological deficit	43 (16.1)	72 (12.2)	0.123
Post–PS match			
No. of patients	199	199	
Mean age in years (range)	55.4 (25–85)	55.6 (23–86)	0.806
Hunt-Hess grade			
I–III	154 (77.4)	166 (83.4)	0.130
IV–V	45 (22.6)	33 (16.6)	
Fisher			
1–3	141 (70.9)	141 (70.9)	1.000
4	58 (29.1)	58 (29.1)	
Hydrocephalus	53 (26.6)	50 (25.1)	0.731
Lab values			
White blood cell, ×109/L, mean ± SD	11.048 ± 3.997	11.180 ± 4.462	0.756
Glucose (mmol/L), mean ± SD	7.233 ± 2.468	7.132 ± 4.929	0.797
Lactate dehydrogenase (U/L), mean ± SD	229.141 ± 71.352	185.633 ± 38.186	<0.001
LDH-phosphate Ratio, mean ± SD	341.684 ± 153.528	189.008 ± 26.814	<0.001
Complication			
Pneumonia	61 (30.7)	60 (30.2)	0.913
Delay ischemic neurological deficit	26 (13.1)	22 (11.1)	0.538

The rIA patients were categorized according to the 3-month outcome (LDH-phosphate Ratio ≥ 226.25 vs. LDH-phosphate Ratio < 226.25). Values are the number of patients (%) or median ± SD or median(range).

## Data Availability

Not applicable.
